# Mechanism for efficient nitrogen utilization by peach rootstock GF677 (*Prunus amygdalus* × *Prunus persica*) in alkaline orchard soils

**DOI:** 10.3389/fpls.2025.1699942

**Published:** 2025-12-01

**Authors:** Hai-yan Song, Ke Zhao, Qiang Wang, Guo-liang Jiang, Shu-xia Sun, Jing Li, Mei-yan Tu, Ling-li Wang, Cheng-yong He, Tie Wang, Dong Chen

**Affiliations:** 1 Horticulture Research Institute, Sichuan Academy of Agricultural Sciences, Chengdu, Sichuan, China; 2 Key Laboratory of Horticultural Crop Biology and Germplasm Creation in Southwestern China of the Ministry of Agriculture and Rural Affairs, Chengdu, Sichuan, China; 3 College of Life Science, Sichuan University, Chengdu, Sichuan, China; 4 Chengdu Agricultural Technology Extension Station, Chengdu, Sichuan, China

**Keywords:** nitrogen utilization, peach rootstock, GF677, alkaline soil, WGCNA

## Abstract

GF677 is a widely used peach rootstock with resistance to chlorosis and continuous-cropping disease. Peach varieties grafted onto GF677 generally exhibit larger leaf area and deep green foliage in alkaline soils, demonstrating a higher nitrogen utilization efficiency (NUE) than other rootstocks. To elucidate the mechanism for the higher NUE of GF677, we grafted four peach varieties onto either GF677 or peach (*Prunus persica*, the control rootstock) in alkaline soils. The results revealed that grafting of peach varieties onto GF677 resulted in larger leaf area and significantly higher total nitrogen content than grafting onto the control rootstock. Comparative transcriptomic analysis and WGCNA showed that the grafting onto GF677 significantly up-regulated 13 genes related to nitrogen uptake, transport, and reuse, while grafting onto the control rootstock up-regulated two monothiol glutaredoxin-encoding genes, in the leaves of different peach varieties. RT-qPCR further confirmed that four genes, including *PpNRT1*, *PpNRT4*, *PpGLDH*, and *PpGRF9*, may play crucial roles in the nitrogen utilization of GF677. Additionally, *PpMGrx2* may play an important role in the response of peach (*Prunus persica*) to alkaline stress. This study elucidates the mechanism underlying the efficient nitrogen utilization of GF677, providing an important basis for the application and improvement of peach rootstock varieties.

## Introduction

According to statistics from the Food and Agriculture Organization of the United Nations (FAO), there are approximately 900 million hectares of saline-alkali soils worldwide, accounting for 7% of all arable soils (Yang and Guo, 2023). Meanwhile, due to the residual salts in irrigation water, the problem of soil salinization is becoming increasingly severe on a global scale (Liang et al., 2024). Among these issues, the damage caused by alkali stress to plants far exceeds that caused by salt stress, manifesting in problems such as nutrient deficiency disorders resulting from reduced nutrient availability (Wei et al., 2021), destruction of soil structure (Song et al., 2025), ionic toxicity (Wang et al., 2025), the inhibition of soil microbial activity (Li D. P. et al., 2025) and so on. The Longquan Mountains area is an important peach producing region in the Sichuan Basin. The soil in the peach orchards of this region was predominantly derived from the weathering of purple shale and therefore has a relatively high calcium carbonate content, which finally results in high soil pH levels (Tu et al., 2018). As a result, chlorosis and continuous-cropping disease have become increasingly prevalent and severe in older peach orchards in this region in recent years, which has brought a serious impact on the yield and fruit quality of the peach orchards (Chen et al., 2018).

The results of previous studies have shown that microbial diversity in the alkaline purple soil of the Sichuan Basin has decreased (Sheng et al., 2023), along with a reduction in the availability and an intensification of the loss of nutrient elements such as carbon (Liu et al., 2025a), nitrogen (Peng et al., 2025), and phosphorus (Song et al., 2015). Notably, chlorosis in perennial fruit trees like peaches, plums, and cherries growing in alkaline purple soil is far more pronounced than in annual crops such as rice and corn (Liu et al., 2022; He et al., 2023). Over the past 15 years, we have compared the relationships between the degree of leaf chlorosis in peach trees and the nutrient elements and physicochemical indicators of the soils. The findings indicate that the contents of bicarbonate ions and carbonate ions in alkaline purple soils are significantly higher than those in normal soils, while the contents of total nitrogen and total iron are lower, ultimately leading to chlorosis in the leaves of peach trees (Chen et al., 2014; Tu et al., 2014, 2018). Given the extremely high cost of soil improvement and the severe continuous-cropping disease that arises from replanting peach (*Prunus persica*) *in situ*, selecting resistant specialized rootstocks has become an inevitable approach to addressing chlorosis in peach orchards in the Sichuan Basin.

Peach trees in production are all combinations of rootstocks and scions, where the rootstock has significant influence on scion growth and fruit quality (Wang, 2021). Peach (*Prunus persica*) is a widely utilized peach rootstock in southern China, primarily employing seed-propagated seedlings as rootstocks. It possesses characteristics such as tolerance to infertile soil and strong graft compatibility but exhibits relatively weak resistance to cold and alkalinity (Jiang, 2023). GF677 (*Prunus amygdalus* × *Prunus persica*), a rootstock with strong alkaline and continuous-cropping resistance, is characterized by a well-developed root system, vigorous growth, a large canopy, and a high grafting compatibility peach (Molassiotis et al., 2006; Gullo et al., 2014; Javed Tareen et al., 2022; Zhang et al., 2023). In 2003, we introduced the peach rootstock GF677 through the Vivai Battistini Company from Italy, which demonstrates some highly desirable characteristics such as resistance to iron-deficiency chlorosis, continuous-cropping disease, and drought in actual production (Li et al., 2008; Song et al., 2018). Results from numerous early studies carried out in normal soils or saline-alkali soils indicated that GF677 demonstrated higher indicators, such as total leaf area (Fallah et al., 2025), total root length (Zrig et al., 2011), and above-ground and below-ground biomass (Gharbi et al., 2025), compared to other rootstocks. Previous researchers ascribed these findings to GF677’s high photosynthetic efficiency, or, to put it briefly, its strong stress resistance (Jimenez et al., 2020). Recently, the results of a grafting experiment conducted in normal soil (which eliminated the influence of GF677’s leaves) revealed that the growth indicators of scion varieties grafted onto GF677 outperformed those grafted onto rootstocks like Penta (a widely used peach rootstock in Europe), which indicated that GF677 might also possess significant advantages in nutrient use efficiency (Gullo et al., 2014).

Our recent studies have revealed that in alkaline soils, genes related to the transport and storage of ferrin in the root system of GF677 are significantly enriched and up-regulated, while the differences in most alkaline tolerance genes between GF677 and peach are not significant (Sun et al., 2020; Song et al., 2023). Additionally, genes associated with nitrogen metabolism and photosynthesis may also be potential reasons for GF677’s resistance to chlorosis (Sun et al., 2022). Although numerous existing field trials suggest that GF677 may outperform other rootstock varieties in terms of nitrogen utilization efficiency (Macci et al., 2016), currently, there are few studies on the nitrogen utilization efficiency (NUE) of GF677, particularly a lack of studies on the NUE of different scion varieties after grafting onto GF677.

Nitrogen is the “backbone element” of chlorophyll, contributing 40%–50% to fruit tree yield, and also an essential component of plant proteins, nucleic acids, and phospholipids (Xu et al., 2012). In plants, nitrogen metabolism initiates with the uptake of inorganic nitrogen (NO_3_^-^ and NH_4_^+^) and small-molecule organic nitrogen through the roots, which is then transported upward via the xylem to the leaves (Tegeder and Masclaux-Daubresse, 2018). After assimilation in the leaves, these nitrogen sources are synthesized into biological macromolecules such as proteins and nucleic acids (Liu et al., 2025b), and some organic nitrogen is subsequently redistributed through the phloem. Genes such as *nitrate transporter* (*NRT*), *nitrite reductase* (*NiR*), *amino acid permease* (*AAP*), *ammonium transporter* (*AMT*), *glutamine synthetase isoenzymes* (*GLN*), and *growth-regulating factor* (*GRF*) play crucial roles in this complex and precisely regulated process (Munos et al., 2004; Gao et al., 2020; Yang et al., 2023; Cai et al., 2024; Zhang et al., 2025).

As a continuation of previous research, we grafted four different peach varieties onto either GF677 (*Prunus amygdalus* × *Prunus persica*) or peach (*Prunus persica*) rootstocks in an old peach orchard with alkaline soils, and then compared the growth indices of the rootstocks and scions, as well as the chlorophyll components and major nutrient indicators in the leaves after grafting. Subsequently, we identified the key genes related to the NUE of GF677 in alkaline peach orchard soils through transcriptome sequencing and weighted gene co-expression network analysis (WGCNA). The findings are expected to lay a solid foundation for research on the NUE in peaches and also facilitate the popularization and application of the GF677 rootstock.

## Materials and methods

### Plant materials and growth conditions

The tested rootstocks were one-year-old seedlings of GF677 (*Prunus amygdalus* × *Prunus persica*) and peach (*Prunus persica*, abbreviated as PP), which were planted in April 2022. In February 2023, four different peach varieties (Huangyu No.1 (HY), Zhongtao No.9 (ZT9), Baifeng (BF), and Wanhujing (WHJ)) were grafted onto the rootstocks of GF677 or PP, which involved fifteen plants for each variety that were arranged into three replicates. The corresponding combinations after grafting were designated as HY/GF677, ZT9/GF677, BF/GF677, WHJ/GF677, HY/PP, ZT9/PP, BF/PP, and WHJ/PP. The experimental site was located in Longquanyi District, Chengdu City, Sichuan Province (E104°24′ 15.95″, N30°37′52.82″, altitude 799 m), where peach trees had been planted for 22 years before. The major chemical properties and mineral nutrients in the soil were pH (dimensionless) 8.47, organic matter content 6.49 g/kg, total nitrogen content 0.040%, total phosphorus content 0.57 g/kg, total potassium content 22.5 g/kg, available phosphorus content 4.7 mg/kg, available potassium content 161 mg/kg, and alkali-hydrolyzable nitrogen content 20 mg/kg. All tested plants were trained in a central leader tree form, with a plant spacing of 1.5 m × 4 m. They were cultivated on raised beds with soil accumulation with conventional management measures.

### Measurement of phenotypic indicators of plants

On April 10, 2023, 10 one-year-old branches with similar growth vigor for each tree were randomly selected and tagged from different orientations on the outer periphery of the tree crown, and their lengths and basal diameters were recorded. Moreover, the diameters at 2 cm above and 2 cm below the grafting joint were also recorded. Subsequently, on September 28, 2023, the aforementioned indicators were measured again. On July 25, 2023, 20 mature functional leaves were randomly collected from each variety to determine the leaf length, width, and aspect ratio. The data for the aforementioned phenotypic indicators were obtained using a tape measure and a digital caliper (DL90150, Deli Group Co., Ltd., Ningbo, Zhejiang, China). All the aforementioned experiments were repeated three times. All data were analyzed for significant differences and plotted using the Chiplot online platform (https://www.chiplot.online/). The collected leaves were transferred with liquid nitrogen and stored in a –80 °C freezer for the determination of other physiological indicators and transcriptome analysis.

### Measurement of chlorophyll and major nutrient element indicators in leaves

The chlorophyll content in leaves was determined using the acetone method. In brief, 0.2 g of leaf tissue was weighed, ground into a fine powder, and then mixed with acetone. The mixture was kept in the dark at 4°C. After 48 h, the extracted solution was transferred to a 25-mL volumetric flask and made up to the mark. The absorbance values at 646 nm and 663 nm were then measured. The determination of the contents of macroelements and trace elements in peach leaves was carried out with reference to previous studies (Zhang et al., 2022; Chen et al., 2021). In brief, the total nitrogen (N) content in leaves was determined using a Fully automatic Kjeldahl nitrogen analyzer (Kjeltec 8400, Food and Agricultural Science Solutions, Denmark). The total potassium (K) content was measured by flame atomic absorption spectrometer (ContrAA300, Analytik Jena, Germany) and the total phosphorus (P) content was determined by ammonium molybdate spectrophotometry. The determination of magnesium (Mg) content in leaves was conducted using inductively coupled plasma mass spectrometry (ICP-MS) (NexION 300x, Perkin Elmer, America). The zinc (Zn) content was measured using dithizone colorimetry, and iron (Fe) content was determined using o-phenanthroline colorimetry. Ten leaves were mixed together to constitute one biological replicate, and all experiments were set up with three replicates. All data were analyzed for significant differences and plotted using the Chiplot online platform (https://www.chiplot.online/).

### Transcriptome sequencing and gene differential expression analysis

The aforementioned leaf samples were used for total RNA extraction. Total RNA was extracted from the samples using the Trizol method. The integrity of RNA was assessed by agarose gel electrophoresis, and its concentration and purity were determined using a Nanodrop One (Thermo Fisher Scientific, Massachusetts, America). RNA samples that passed quality control were used for library construction and transcriptome sequencing. Low-quality sequences, adapter sequences, and sequences containing unknown bases were removed. Gene expression levels were represented by Fragments Per Kilobase of exon model per Million mapped fragments (FPKM) values. Analysis of differentially expressed genes (DEGs) was performed using DESeq2 (Love et al., 2014) with |Log_2_ fold change| ≥ 1 and *p*-value < 0.05. Gene Ontology (GO) enrichment analysis was performed using the topGO R package (Song et al., 2024). The annotation of DEGs was obtained using the Kyoto Encyclopedia Genes and Genomes (KEGG) database.

### Screening of key genes related to nitrogen accumulation with WGCNA

Weighted gene co-expression network analysis (WGCNA) was performed using the cloud platform of Metaware Company (https://cloud.metware.cn/), where the Pearson correlation coefficient was calculated for the transcriptome data, chlorophyll (Chl) content, and six major nutrient elements. Subsequently, based on the correlation with total nitrogen content, the two modules exhibiting the highest positive correlations were identified.

### Verification of key gene expression patterns via quantitative real-time PCR

Based on the gene annotations, 13 genes associated with nitrogen metabolism and transport, along with two genes encoding monothiol glutaredoxin-S1 related to stress resistance, were selected for subsequent validation using quantitative real-time PCR (qRT-PCR). The total RNA was extracted using the Plant Total RNA Extraction Kit (DP441, TIANGEN, Beijing, China), and qRT-PCR was performed using the blastaqTM 2× qPCR Master Mix Kit (G891, ABM, Vancouver, Canada). All experiments were carried out in accordance with the respective operation manuals. The list of all primers is provided in **Supplementary Table S1**.

## Results

### Growth performance of four peach varieties grafted onto GF677 or peach (*Prunus persica*)

**Figure 1:**
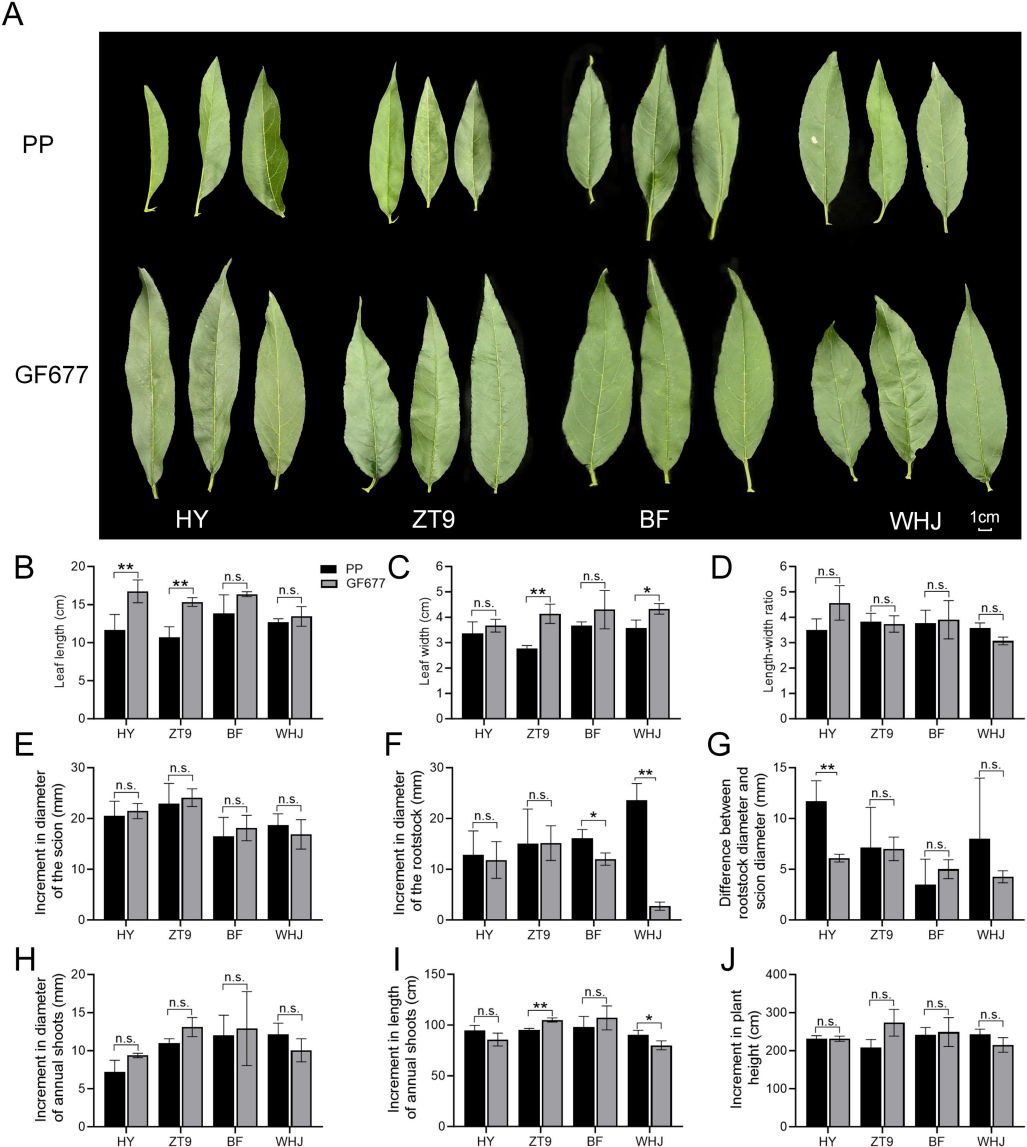
Growth performance of four peach varieties grafted onto GF677 or peach (*Prunus persica*) rootstocks. **(A)** Phenotype of leaves. Bar = 1 cm. **(B–D)** Comparison of leaf length, width, and length-to-width ratio. **(E, F)** Comparison of the diameter increments of the scion and rootstock within half a year after grafting. **(G)** Difference between rootstock diameter and scion diameter half a year after grafting. **(H, I)** Increment in diameter and length of annual shoots half a year after grafting. **(J)** Increment in plant height half a year after grafting. One or two asterisks indicate statistical significance according to Student ‘s *t*-test at the 0.05 or 0.01 level, respectively.

As shown in **Figure 1A**, peach varieties grafted onto GF677 had significantly larger mature functional leaves than those grafted onto peach rootstock PP. Specifically, HY/GF677 (scion/rootstock, the same below) and ZT9/GF677 showed extremely significantly longer leaf lengths than HY/PP and ZT9/PP, respectively (**Figure 1B**). Moreover, ZT9/GF677 and WHJ/GF677 had significantly or extremely significantly wider leaf widths than ZT9/PP and WHJ/PP, respectively (**Figure 1C**). However, grafting onto GF677 and PP resulted in no significant differences in the aspect ratio (length-to-width ratio) of leaves in all tested varieties (**Figure 1D**).

Incompatibility between the rootstock and scion of fruit trees may lead to different growth rates above and below the graft union, ultimately resulting in a phenomenon of ‘big feet’ or ‘small feet’ (Amri et al., 2021). We recorded the diameter increments of the upper part (represented by the scion) and lower part (represented by the rootstock) of the graft union over a six-month period for plants grafted onto GF677 or PP, respectively (**Figures 1E–G**). The results showed that grafting onto GF677 and PP resulted in no significant differences in the diameter increment of the scions in all tested varieties. However, BF/PP and WHJ/PP showed significantly or extremely significantly higher diameter increments of the rootstock than BF/GF677 and WHJ/GF677, respectively (**Figure 1F**). Notably, HY/PP showed extremely significantly greater difference between the rootstock diameter and the scion diameter than HY/GF677 (**Figure 1G**).

In addition, follow-up observation revealed that grafting onto GF677 and PP led to no significant differences in diameter increment at the base of one-year-old shoots (**Figures 1H–J**). ZT9/GF677 exhibited extremely significantly higher increment in shoot length than ZT9/PP. Conversely, WHJ/PP showed a significantly greater increment than WHJ/GF677 (**Figure 1I**). Furthermore, there were no significant differences in the increment of plant height between GF677 and PP grafting (**Figure 1J**).

### Changes in chlorophyll and major nutrient elements in peach leaves after grafting

**Figure 2:**
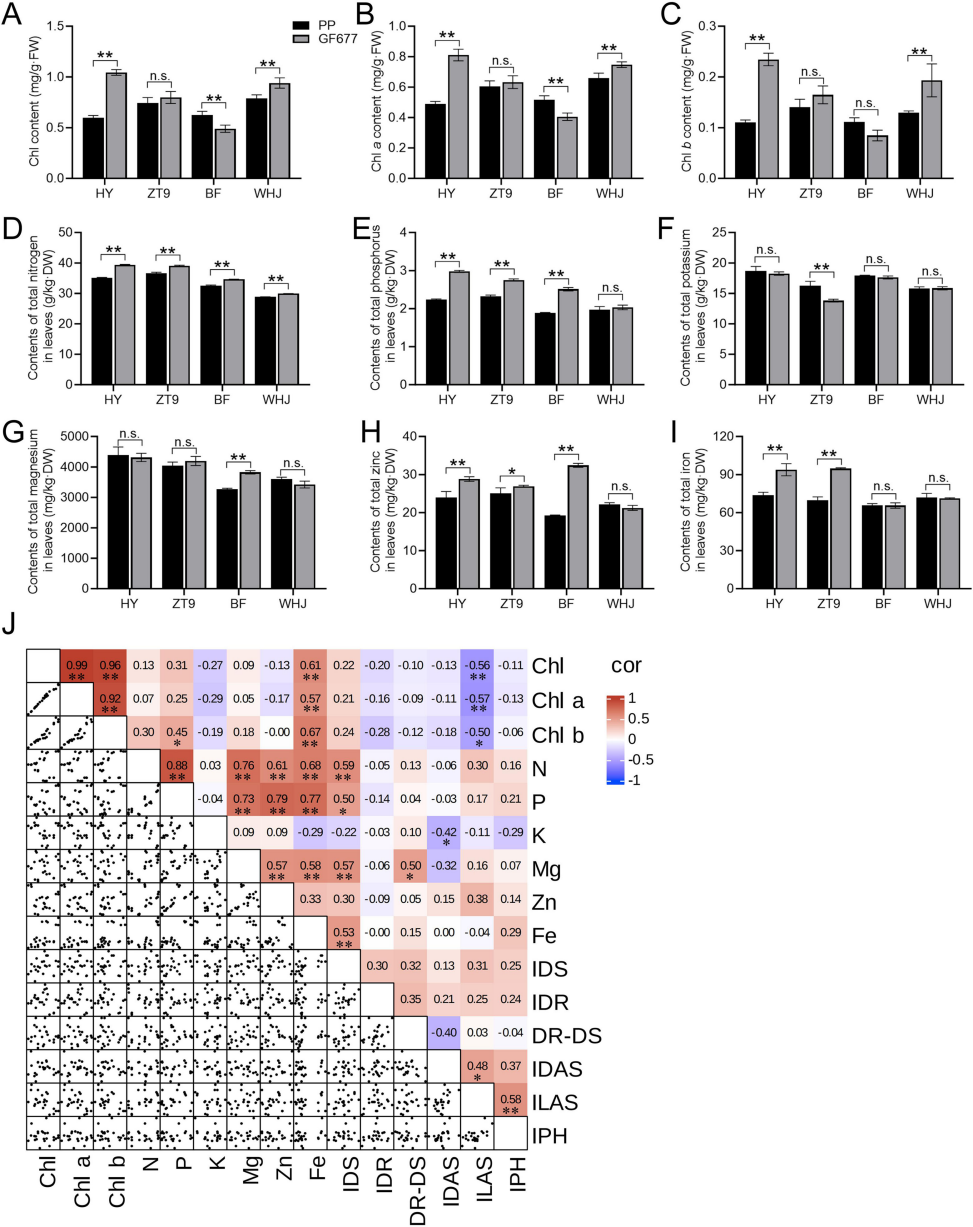
Changes in chlorophyll and major nutrient element contents in peach leaves after grafting. **(A–C)** Comparison of the contents of total chlorophyll **(A)**, chlorophyll a **(B)**, and chlorophyll b **(C)** in leaves. **(D–I)** Comparison of the contents of total nitrogen **(D)**, phosphorus **(E)**, potassium **(F)**, magnesium **(G)**, zinc **(H)**, and iron **(I)** in leaves. **(J)** Pearson correlation coefficients between the chlorophyll content, major nutrient element content in the leaves, and plant growth indicators across all samples. Chl: Total chlorophyll, Chl a: chlorophyll a, Chl b: chlorophyll b, N: nitrogen, P: phosphorus, K: potassium, Mg: magnesium, Zn: zinc, Fe: iron, IDS: increment in diameter of the scion, IDR: increment in diameter of the rootstock, DR-DS: difference between rootstock diameter and scion diameter, IDAS: increment in diameter of annual shoots, ILAS: increment in length of annual shoots, IPH: increment in plant height. One or two asterisks indicate statistical significance according to Student ‘s *t*-test at the 0.05 or 0.01 level, respectively.

HY/GF677 and WHJ/GF677 had extremely significantly higher contents of chlorophyll a, chlorophyll b, and total chlorophyll in the leaves than HY/PP and WHJ/PP, respectively (**Figures 2A–C**). Conversely, BF/GF677 exhibited extremely significantly lower contents of chlorophyll a and chlorophyll b in the leaves than BF/PP (**Figures 2A, B**).

Determination of the major nutrient elements in mature functional leaves revealed that grafting onto GF677 resulted in extremely significantly higher total nitrogen contents in the leaves of all the four peach varieties compared with grafting onto PP (**Figure 2D**). Additionally, HY/GF677, ZT9/GF677, and BF/GF677 had significantly or extremely significantly higher total phosphorus and total zinc contents in the leaves than HY/PP, ZT9/PP, and BF/PP, respectively (**Figures 2E, H**). BF/GF677 showed an extremely significantly higher total magnesium content in the leaves relative to BF/PP (**Figure 2G**). Notably, HY/GF677 and ZT9/GF677 leaves had extremely significantly higher total iron contents than HY/PP and ZT9/PP leaves, respectively (**Figure 2I**).

We further conducted a correlation analysis to examine the relationships between the chlorophyll content, major nutrient element content in the leaves, and plant growth indicators across all samples (**Figure 2J**). The results revealed that the increment in diameter of the scion (IDS) exhibited significant or extremely significant positive correlations with the contents of N, P, Mg, and Fe in the leaves. The difference between rootstock diameter and scion diameter (DR–DS) was significantly positively correlated with the Mg content. The increment in diameter of annual shoots (IDAS) displayed a significant negative correlation with the K content. The increment in length of annual shoots (ILAS) demonstrated significant or extremely significant negative correlations with the contents of chlorophyll a, chlorophyll b, and total chlorophyll, but significant or extremely significant positive correlations with IDAS and increment in plant height (IPH).

### Comparative transcriptome analysis of peach leaves after grafting

**Figure 3:**
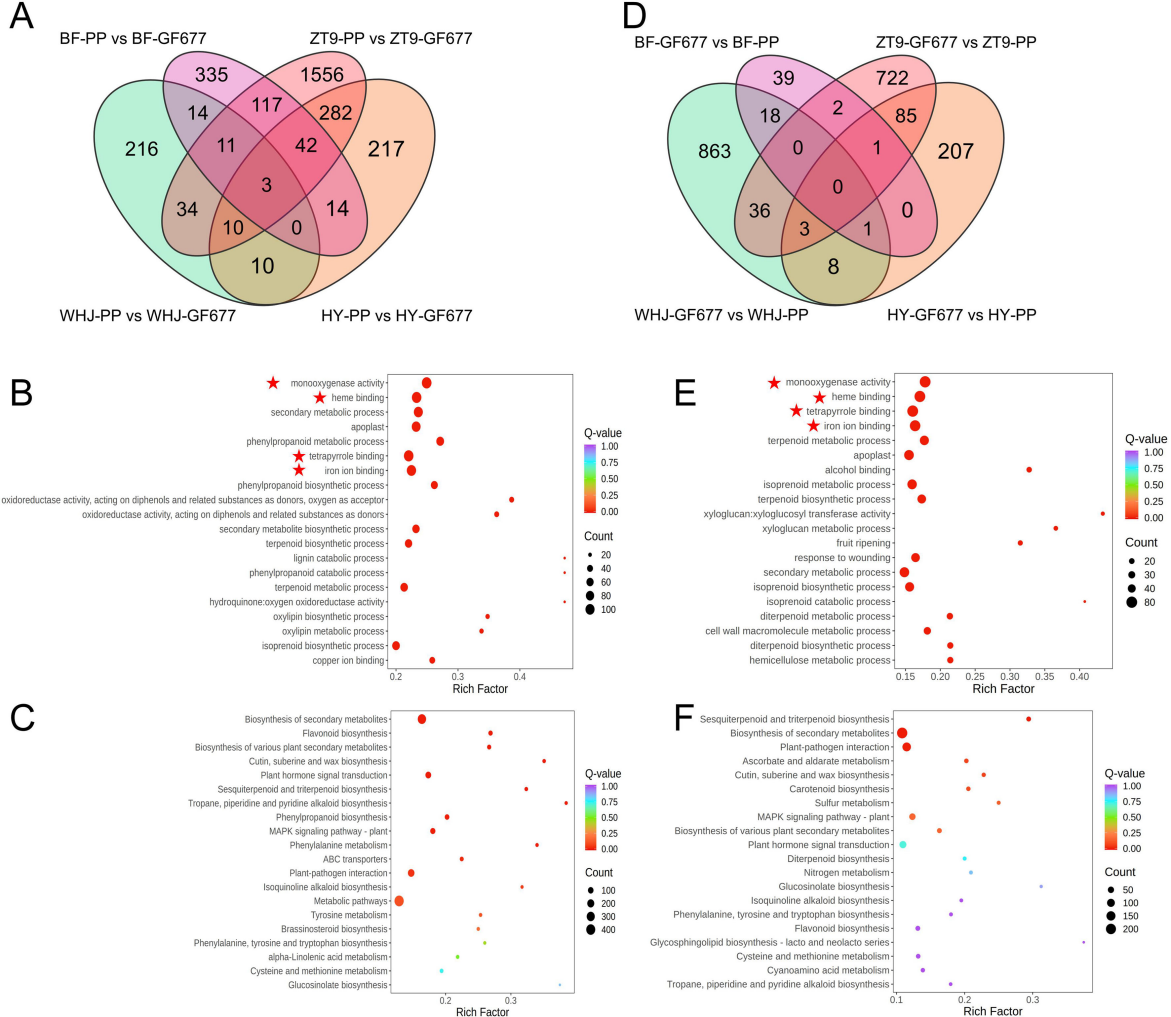
Comparative transcriptome analysis of leaves from four peach varieties grafted onto GF677 or peach (*Prunus persica*) rootstocks grown in alkaline soil. Results of the Venn diagram **(A)**, GO enrichment analysis **(B)**, and KEGG enrichment analysis **(C)** for up-regulated genes in the four peach varieties grafted onto peach (*Prunus persica*) rootstocks. Results of the Venn diagram **(D)**, GO enrichment analysis **(E)**, and KEGG enrichment analysis **(F)** for up-regulated genes in the four peach varieties grafted onto GF677 rootstocks.

The transcriptome analysis results revealed that in the leaves of the four peach varieties grafted onto PP, a total of 2,861 genes were up-regulated (**Figure 3A**). Notably, three genes were commonly up-regulated in all four varieties, and two of them encoded monothiol glutaredoxin. GO enrichment analysis indicated that these genes were enriched in terms related to iron metabolism and stress responses, such as monooxygenase activity, heme binding, tetrapyrrole binding, and iron ion binding (**Figure 3B**). KEGG enrichment analysis showed that these genes were primarily involved in 20 major pathways, including secondary metabolite biosynthesis, plant-pathogen interaction, and plant hormone signal transduction (**Figure 3C**).

In contrast, in the leaves of plants grafted onto GF677, a total of 1,985 genes were up-regulated (**Figure 3D**). GO enrichment analysis indicated that these genes were primarily enriched in terms related to iron metabolism and stress responses, such as monooxygenase activity, heme binding, tetrapyrrole binding, and iron ion binding (**Figure 3E**). The KEGG enrichment results were similar to those obtained with PP as the rootstock. However, some genes were enriched in pathways such as nitrogen metabolism and MAPK signaling pathway (**Figure 3F**).

### Screening of key genes responsive to alkaline soil conditions

**Figure 4:**
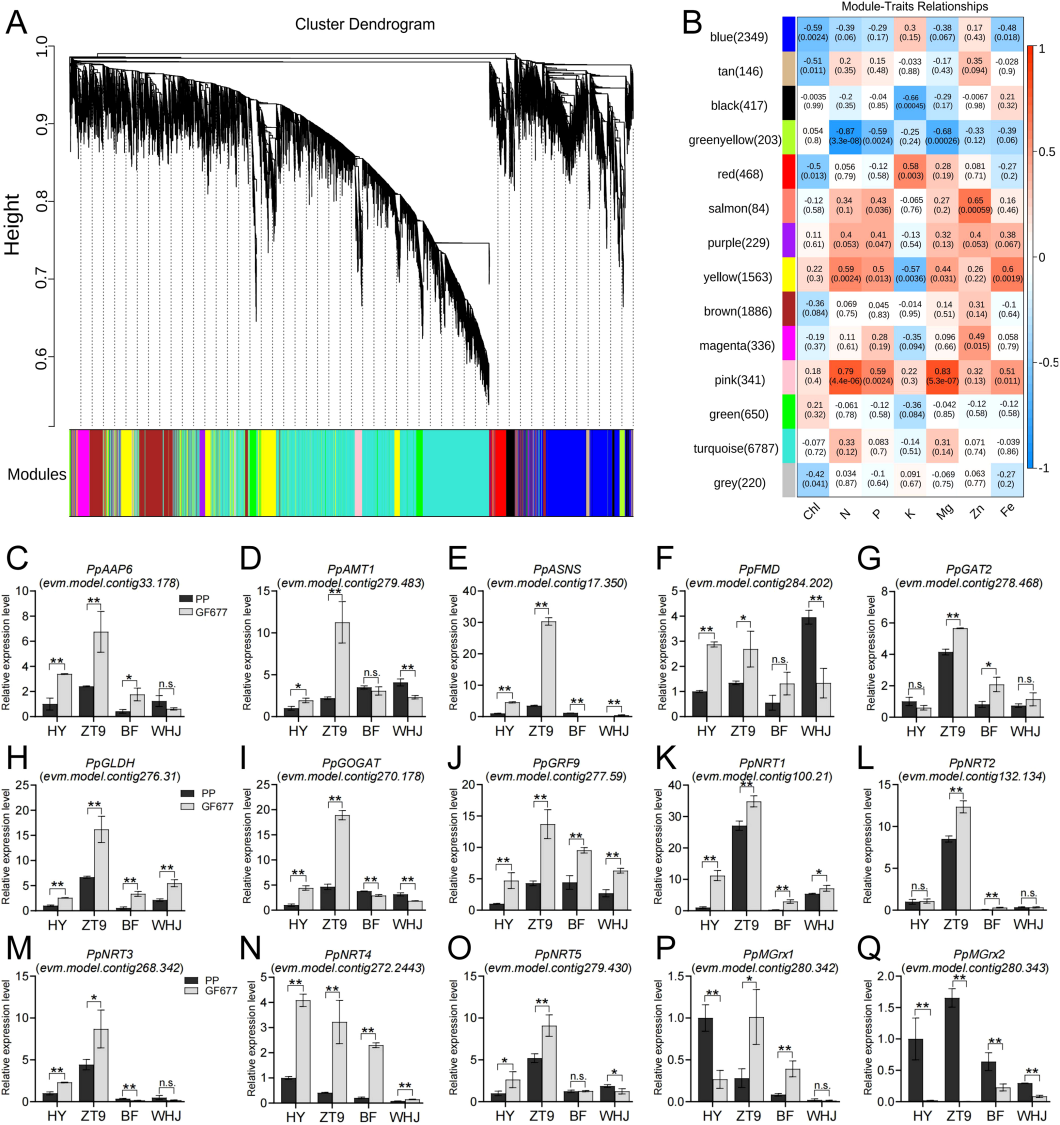
Screening of key genes responsive to alkaline soil conditions based on WGCNA. **(A)** Module hierarchical clustering dendrogram. **(B)** Correlation heatmap between modules and major nutrient indices. **(C–Q)** Results of RT-qPCR for 13 genes involved in nitrogen uptake, transport, and reuse, as well as 2 monothiol glutaredoxin-encoding genes in all rootstock-scion combinations. One or two asterisks indicate statistical significance according to Student ‘s *t*-test at the 0.05 or 0.01 level, respectively.

We conducted WGCNA on the transcriptome data and major nutrient indices of leaves from all rootstock-scion combinations (**Figures 4A, B**). The results revealed that the pink and yellow modules exhibited highly positive correlations with the contents of N, P, Mg, and Fe. A total of 13 genes involved in nitrogen uptake, transport, and reuse were screened from these two modules. Subsequently, we performed RT-qPCR validation on these 13 genes and two monothiol glutaredoxin-encoding genes. Grafting onto GF677 led to significantly or extremely significantly higher expression levels of *PpGLDH*, *PpGRF9*, *PpNRT1*, and *PpNRT4* in all four varieties than grafting onto PP. However, an exactly opposite result was obtained for the *PpMGrx2* gene. Furthermore, the RT-qPCR results were highly consistent with the transcriptome data, confirming the reliability of the transcriptome sequencing.

## Discussion

The interaction between rootstock and scion represents one of the most crucial relationships in fruit tree production (Hayat et al., 2021). The rootstock significantly influences the traits such as growth vigor (Li et al., 2024), yield (Li B. X. et al., 2025), fruit quality (Saeed et al., 2024), and resistance of fruit trees (He et al., 2024) by regulating the root system architecture, nutrient uptake, water use efficiency, and hormone balance. Our previous studies have indicated that the root of GF677 exhibits stronger iron translocation and accumulation capabilities, thereby conferring strong resistance to iron-deficiency chlorosis (Sun et al., 2020, 2022). Furthermore, GF677 was also found to advance the flowering period and enhance the yield of scion varieties (Pica et al., 2021). In this study, grafting of four peach varieties onto GF677 resulted in larger leaf area and higher total nitrogen content, demonstrating higher NUE of the rootstock. Also, there were notable differences in the performance of different scion varieties when grafted onto GF677. Among the 18 plant growth indicators, HY/GF677, ZT9/GF677, BF/GF677, and WHJ/GF677 outperformed plants grafted onto PP in 9, 7, 4, and 5 indicators, respectively, with significant or extremely significant differences. Conversely, HY/GF677, ZT9/GF677, BF/GF677, and WHJ/GF677 underperformed plants grafted onto PP in 0, 1, 3, and 2 indicators, respectively, with significant or extremely significant differences. Overall, after grafting onto GF677, the growth performance of the four peach varieties followed the order of HY/GF677 > ZT9/GF677 > WHJ/GF677 > BF/GF677. These findings provide an important theoretical basis for future selection of suitable rootstocks for peach trees.

A study of the NUE of two different rootstocks in normal soils of peach orchards revealed that GF677 could absorb more soil nutrients than Franco (a widely used peach rootstock in Europe), thereby contributing to greater growth and higher yield (Macci et al., 2016). In this study, in alkaline orchard soils, grafting onto the GF677 rootstock resulted in significant up-regulation of genes related to nitrogen metabolism, monooxygenase activity, heme binding, tetrapyrrole binding, and iron ion binding in the leaves of scions. Among them, *PpGLDH*, *PpGRF9*, *PpNRT1*, and *PpNRT4* exhibited an average up-regulation of 3.41, 3.10, 5.69, and 6.20 folds, respectively, in the leaves after GF677 grafting. Our research has once again provided strong evidence for the universality of GF677’s high nitrogen use efficiency (NUE) in both normal soils and alkaline soils. Future dynamic observation of the various forms of nitrogen and related enzyme activities in the soil, roots, and leaves will help to comprehensively elucidate mechanism for efficient nitrogen utilization in GF677.

Nitrate transporters (NRT) can not only participate in nitrate uptake and transport in plants, but also play key roles in many other physiological processes, such as development of the root system (Lee et al., 2024), uptake and transport of other mineral ions through hormone transport (Liu et al., 2025c), signal transduction (Hu et al., 2019), and even the transport of other ions (Ma et al., 2025), thereby affecting the plant stress performance related to these ions. Recent research findings indicate that the nitrate transporter (NRT2) and calcium-binding protein (CaBP) gene families in wild barley (*Hordeum brevisubulatum*) have undergone significant expansion, with markedly upregulated expression under alkaline stress (Feng et al., 2025). This has led to the formation of a unique CaBP-NRT2 module capable of enhancing the plant’s nitrogen utilization efficiency, thereby strengthening its alkaline tolerance. Additionally, *GhGLDH35A* and *AtGRF6* also play crucial roles in the alkaline stress resistance processes of cotton (Fan et al., 2025) and Arabidopsis thaliana (Guo J. F. et al., 2022), respectively. Thus, it is evident that improving nitrogen utilization efficiency represents an important strategy for plants to cope with alkaline stress.

**Figure 5:**
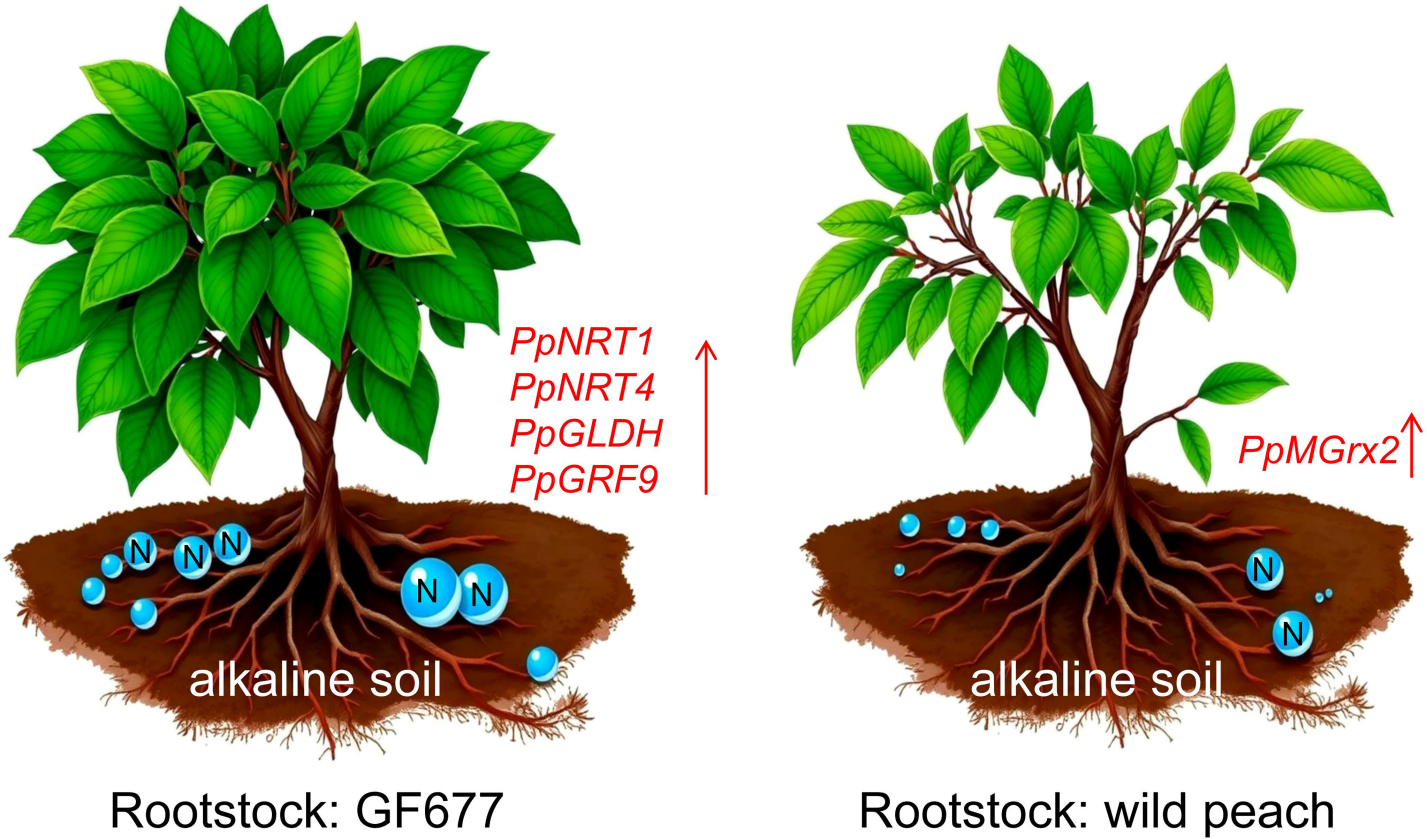
A model depicting nitrogen utilization and the response to alkaline soils in GF677 and peach (*Prunus persica*) rootstocks within peach orchards. *PpNRT1*, *PpNRT4*, *PpGLDH*, and *PpGRF9* may make significant contributions to the high nitrogen use efficiency (NUE) of GF677. *PpMGrx2* may play a crucial role in the peach’s response to alkaline stress.

Monothiol glutaredoxins (Grxs) represent one of the vital intracellular redox systems, which are extensively involved in various processes such as stress resistance responses (Guo X. et al., 2022), reactive oxygen species (ROS) scavenging (Mittler et al., 2022), and iron-sulfur protein maturation (Moseler et al., 2015), and display conservation across different species. Previous research has demonstrated that Grx3 is essential for the growth of *Candida albicans* under low-iron conditions (Alkafeef et al., 2020). In *Azorhizobium caulinodans*, Grxs play a crucial role in combating ROS and in the symbiotic nitrogen fixation process with *Sesbania rostrata* (Cao et al., 2020). Moreover, under low peroxide concentrations, the loss of function of dithiol Grxs can be compensated by monothiol Grxs. In this study, grafting onto PP resulted in significantly higher expression of two monothiol glutaredoxin-encoding genes in the leaf transcriptomes of four peach varieties. Furthermore, RT-qPCR results confirmed that the four varieties grafted onto PP had 2.81–285.18 times higher expression levels of *PpMGrx2* compared with those grafted onto GF677. Therefore, based on these research findings, we propose a model for the nitrogen utilization and response to alkaline soils of GF677 and peach (*Prunus persica*) rootstock in peach orchards (**Figure 5**). In future research, the gene functions of *PpNRT1*, *PpNRT4*, and *PpMGrx2* can be further validated to discover novel targets for enhancing peach resistance to alkaline stress.

## Conclusion

In the study, we grafted four different peach varieties onto either GF677 (*Prunus amygdalus* × *Prunus persica*) or peach (*Prunus persica*) rootstocks in an old peach orchard with alkaline soils, and then compared the growth indices of the rootstocks and scions, as well as the chlorophyll components and major nutrient indicators in the leaves after grafting. The results showed that grafting onto GF677 resulted in larger leaf area and a highly significant elevation in total nitrogen content in the four peach varieties compared with grafting onto peach (*Prunus persica*). Comparative transcriptomic analysis and WGCNA revealed significant up-regulation of 13 genes related to nitrogen uptake, transport, and reuse in GF677, among which four genes, including *PpNRT1*, *PpNRT4*, *PpGLDH*, and *PpGRF9*, may have great contributions to the high NUE of GF677. Additionally, *PpMGrx2* may play an important role in the response of peach (*Prunus persica*) to alkaline stress. This study confirmed the mechanism by which GF677 enhances its adaptability in alkaline soils through improved NUE, and identified four key genes involved in this process, providing important targets for the improvement of peach varieties with higher NUE.

## Data Availability

The datasets presented in this study can be found in online repositories. The names of the repository/repositories and accession number(s) can be found below: https://ngdc.cncb.ac.cn/gsa/, CRA029529.
